# Anxiety and depression symptoms among medical residents in KSA during the COVID-19 pandemic

**DOI:** 10.1016/j.jtumed.2022.01.005

**Published:** 2022-02-05

**Authors:** Hossam S. Alawad, Hussein S. Amin, Eiad A. Alfaris, Abdullah M. Ahmed, Fahad D. Alosaimi, Ahmed S. BaHammam

**Affiliations:** a King Saud University Chair for Medical Education Research and Development, Department of Family and Community Medicine, College of Medicine, King Saud University, Riyadh, KSA; b Psychiatry Department, College of Medicine, King Saud University, Riyadh, KSA; c Department of Medicine, College of Medicine, University Sleep Disorders Centre, King Saud University, KSA; d The Strategic Technologies Program of the National Plan for Sciences and Technology and Innovation in the KSA, King Saud University, Riyadh, KSA

**Keywords:** مقياس اضطراب القلق المعمم, استبانة صحة المريض, الجنس, الصحة العامة, التدريب الطبي, Gender, Generalized anxiety disorder scale, Medical training, Patient health questionnaire, Public health

## Abstract

**Objectives:**

Medical residents’ direct contact with patients with COVID-19 places them at high risk of psychological disturbance. This study aimed to estimate the prevalence of depression and anxiety symptoms, and their relationship with the COVID-19 pandemic among medical residents in KSA.

**Methods:**

A cross-sectional study was conducted between January and March of 2021. The Patient Health Questionnaire (PHQ-9) and the Generalized Anxiety Disorder Scale (GAD-7) were used to screen for depressive disorders and generalized anxiety disorder, respectively.

**Results:**

A total of 533 medical residents participated in the study; 52% were men, and 58% were single. Most residents had direct contact with one or more patients with COVID-19. The prevalence of depression and anxiety symptoms was 65.8% and 58.3%, respectively. The study revealed that gender was a risk factor for diagnosis with COVID-19 among residents: male residents were diagnosed with COVID-19 to a greater extent than female residents. In addition, being a nonsmoker rather than a smoker was associated with a higher risk of COVID-19 diagnosis. A multivariate regression analysis revealed that gender (female) and residency level (R5) were independently associated with anxiety symptoms. Similarly, the independent correlates of depression symptoms were gender (female) and specialty (family medicine).

**Conclusion:**

A high prevalence of depression and anxiety symptoms was found among medical residents trained in KSA. The rates were significantly higher among female than male residents.

## الملخص

**أهداف البحث:**

يحتك الأطباء المقيمين مع مرضى فيروس كورونا المستجد بشكل مباشر مما يجعلهم عُرضة للاضطرابات النفسية. تهدف الدراسة لاستنتاج نسبة انتشار الاكتئاب والقلق بين الأطباء المقيمين في المملكة العربية السعودية وعلاقتهما بجائحة فيروس كورونا المستجد.

**طرق البحث:**

أُجريت هذه الدراسة خلال الفترة من يناير 2021 إلى مارس 2021. تم استخدام استبانة صحة المرضى لفحص الاكتئاب وكذلك مقياس اضطراب القلق العام لفحص القلق.

**النتائج:**

شارك 533 طبيب مقيم في هذه الدراسة ‏وقد بلغ متوسط أعمارهم 28.47±2.90 ‏ ‏وكان غالبيتهم سعوديي الجنسية ومثل الذكور 52% من العدد الكلي والعزاب 58%. معظم الأطباء المقيمين تعرضوا لاحتكاك مباشر مع مريض أو أكثر مصاب بفيروس كورونا المستجد. بلغت نسبة انتشار الاكتئاب 65.8% وأما القلق 58.3%. كشفت الدراسة أن أعلى عوامل الخطورة في الإصابة بفيروس كورونا المستجد هي عند الأطباء المقيمين الذكور وكذلك غير المدخنين. هناك ارتباط معتبر بين القلق وكل من الأطباء المقيمين النساء والأطباء المقيمين في المستوى الخامس من التدريب. أيضا هناك ارتباط معتبر بين الاكتئاب وكل من الأطباء المقيمين النساء والتخصصات (تخصص طب الأسرة).

**الاستنتاجات:**

وجد أن هناك نسبة عالية من الاكتئاب والقلق بين الأطباء المقيمين في المملكة العربية السعودية. وكانت النسبة أعلى بشكل معتبر عند الأطباء المقيمين النساء.

## Introduction

Coronavirus disease 2019 (COVID-19) is caused by a novel virus, SARS-CoV-2, discovered in 2019. The illness can affect multiple organ systems in humans, thus leading to increased morbidity and mortality. In addition, this disease has been repeatedly shown to significantly affect mental health. Previous studies on severe acute respiratory syndrome (SARS) and Ebola have revealed a tendency toward more severe emotional distress during the outbreaks of such epidemics.^1^ These psychological consequences are not only short-term during the pandemic but can extend for some time after the infection.^2^^,^^3^

SARS, Middle East respiratory syndrome, and COVID-19 have been found to substantially affect the physical and mental health of healthcare workers.^4^ Depression and anxiety symptoms have been prevalent in KSA and the Gulf region during the COVID-19 pandemic.^5^^,^^6^ Residents are doctors in training who practice medicine under the supervision of attending physicians. During residency training, residents have high workloads and long working hours. They take care of patients, may provide on-call coverage, and take exams. Being a resident doctor rather than another hospital worker is associated with clinical anxiety.^7^ In addition, frontline medical staff are more likely to have psychological disturbances than the general population.^8^

Furthermore, frontline medical workers have higher rates of anxiety symptoms and depressed mood than nonfrontline medical workers.^9^ Medical residents have been compared to soldiers on the frontline during the combat of the COVID-19 pandemic; they have been in direct contact with patients with COVID-19 at their training hospitals, thereby increasing stress levels and workloads.^10^ Being in contact with patients with COVID-19 is a relevant predictor of anxiety and depression.^11^ Data on anxiety and depression symptoms among medical residents in KSA during the COVID-19 pandemic are limited, despite the tremendous need for such information.

Therefore, we hypothesized that anxiety and depression symptoms are prevalent among medical residents in KSA. Understanding this prevalence might aid in identifying vulnerable medical residents to develop screening and early interventions to protect them, and to develop clear policies to ease stress and anxiety among medical residents in future epidemics and pandemics. Therefore, this study aimed to estimate the prevalence of both anxiety and depression symptoms, and to determine the relationships of anxiety and depression symptoms with COVID-19 among medical residents in KSA.

## Materials and Methods

### Study population

This cross-sectional study comprised medical residents registered with the Saudi Commission for Health Specialties (SCFHS), the organization responsible for supervising and evaluating training programs and setting controls and standards for the practice of health professions.^12^ The target population included all medical residents enrolled in Saudi Arabian residency programs (13,537 residents). Postgraduate dental program residents were excluded. The study was conducted between January and March of 2021.

### Recruitment

The SCFHS was officially contacted to help send the online questionnaire link through simple random sampling to a group of residents’ emails. We randomly targeted 2000 residents, then chose every third number in the list of the residents for the final sample (n = 660). We received complete responses from 533 residents, with a response rate of approximately 81%. The link sent to residents included the study title and purpose, questionnaire content, information on privacy and confidentiality, expected time to complete the survey (10 min), and informed consent.

#### Sample size

The sample size was calculated with a confidence interval (CI) of 95% and a 5% margin of error with a prevalence of anxiety and depression symptoms (60.1% and 66.6%, respectively),^10^ with a diagnostic threshold of ten or more, on the basis of a recent study among residents in KSA during the COVID-19 pandemic.^10^ The estimated minimum sample size required was 369.

#### Data collection tool

A self-reported online questionnaire was used that collected information regarding demographic characteristics (such as age, gender, nationality, and marital status), workload (such as on-call coverage), lifestyle (such as exercise, smoking, and alcohol consumption), and medical history (such as current or past history of medical or psychiatric illnesses), and included questions about COVID-19 (e.g., regarding treating patients diagnosed with COVID-19).

#### Instrument

The Patient Health Questionnaire (PHQ-9) is a reliable and valid measure of depression severity and criteria-based diagnosis of depressive disorders.^13^ The Generalized Anxiety Disorder Scale (GAD-7) is a valid and efficient tool for screening GAD and assessing its severity.^14^ A cut-off score ≥10 indicates a possible diagnosis of depression with a sensitivity of 89% and specificity of 82%, whereas GAD has a sensitivity of 85% and specificity of 89%.^14^^,^^15^ English versions of the questionnaires were used, because medicine is taught in English in KSA.

### Validation of the data collection tool

Four senior consultant experts in psychiatric research reviewed the questionnaire (not including the PHQ-9 and GAD-7). A pilot study was conducted on 20 residents, and according to their feedback, the questionnaire was edited.

### Statistical analysis

Data were analyzed in SPSS Pc + version 21.0 statistical software. Descriptive statistics (mean, standard deviation, frequencies, and percentages) were used to describe the quantitative and categorical variables. Chi-square tests were used to compare categorical variables. Analysis of variance was used for variables with more than two factors, whereas Tukey's tests were used to make post hoc test comparisons.

A univariate logistic regression analysis was conducted wherein one independent variable was tested at a time in the model to assess the associations among demographic characteristics, training level, and specialty with anxiety and depression symptoms. Variables with significant p values were included in the multivariable logistic regression analysis model. The correlation matrix assessed the multicollinearity between variables in the model, and no multicollinearity was detected. Moreover, the standard error in the model was used to reassess multicollinearity. A p-value <0.05 was considered statistically significant.

## Results

A total of 533 residents participated in the study; the mean (±SD) age was 28.47 (±2.901), with an SE of 0.126 and range of 24–44 years), and 52.3% were men. The majority were of Saudi nationality (97%), and 58.2% were single. Approximately half the respondents were from the central region (51.4%), followed by the western (21%) and eastern regions (18.4%). Most the respondents were living with their families (74.9%). The monthly income of most of the residents (77.3%) ranged between 15,000 SAR and less than 20,000 SAR, and 42.7% were satisfied with their income. The residents included R1 (21.4%), R2 (24.6%), R3 (30%), R4 (18.6%), and R5 (5.4%). The residents worked in the following specialties: family medicine (26.6%), surgical specialties (26.5%), internal medicine (13.3%), pediatrics (10.7%), emergency medicine (6.9%), and other specialties (15.9%). Most residents (71.1%) were on call (Table 1).

**Table 1:** Sociodemographic characteristics of medical residents.

Characteristic	N (%)
**Mean age (SD)**	28.47 (2.9)
**Male gender**	279 (52.3)
**Saudi nationality**	517 (97)
**Marital status**	
Single	310 (58.2)
Married	214 (40.2)
Divorced	7 (1.3)
Widow/widower	2 (0.4)
**Have children**	125 (23.5)
**Region**	
Central	274 (51.4)
Western	112 (21)
Eastern	98 (18.4)
Southern	33 (6.2)
**Northern**	16 (3)
**Live with family**	399 (74.9)
**Monthly income in Saudi riyals**	
Less than 15,000	47 (8.8)
15,000–20,000	412 (77.3)
More than 20,000	74 (13.9)
**Satisfaction with current income**	
Satisfied	228 (42.7)
Not sure	133 (25)
Dissatisfied	172 (32.3)
**Residency year**	
R1	114 (21.4)
R2	131 (24.6)
R3	160 (30)
R4	99 (18.6)
R5	29 (5.4)
**Main specialty**	
Family medicine	142 (26.6)
Surgical specialties	141 (26.5)
Internal medicine	71 (13.3)
Pediatrics	57 (10.7)
Emergency medicine	37 (6.9)
Others	85 (15.9)
**On call as part of work**	379 (71.1)

Approximately one-third (29.5%) of the residents exercised for at least 150 min per week. Approximately one-third (32.8%) of respondents smoked cigarettes, electronic cigarettes, or shisha, whereas 4.7% drank alcohol. Among all respondents, 19.3% had a current or past history of medical illnesses, such as sleep disorders, asthma, and migraines. In contrast, 21.8% of the residents had a current or past history of psychiatric illnesses. These illnesses included anxiety, depression, and obsessive-compulsive disorder. Approximately one-quarter (24.6%) of respondents received professional psychological help. However, most residents (80.3%) had never been educated or trained in stress management and coping with burnout phenomena.

The prevalence of depression and anxiety symptoms was 65.8% and 58.3%, respectively. In addition, approximately one-third (35.1%) of the residents felt that they would be better off dead or hurting themselves in some way in the 2 weeks before completing the questionnaire (Table 2).

**Table 2:** Lifestyle and medical history of residents.

Lifestyle and medical history of residents		N (%)
Exercising at least 150 min per week	Yes	157 (29.5)
Smoking	Yes	175 (32.8)
Alcohol consumption	Yes	25 (4.7**)**
Current or past history of medical illnesses	Yes	103 (19.3)
Current or past history of psychiatric illnesses	Yes	116 (21.8)
Ever received any professional psychological help	Yes	131 (24.6)
Ever educated or trained in stress management or experienced burnout phenomena	Yes	105 (19.7)
Depression (based on PHQ-9)	Yes	303 (56.8)
GAD (based on GAD-7)	Yes	311 (58.3)
Thoughts of being better off dead or of hurting oneself	Yes	187 (35.1)
Thoughts of being better off dead or of hurting oneself		
Not at all		346 (64.9)
Several days		90 (16.9)
More than half the days		55 (10.3)
Nearly every day		42 (7.9)

Most residents (80.4%) jobs required them to treat patients diagnosed with COVID-19. Approximately half the respondents (53.8%) had been isolated because of suspected SARS-CoV-2 infection. Additionally, half the respondents (50.5%) had been isolated two times or more. Approximately one-fifth (18.6%) of the residents had been diagnosed with COVID-19.

The most common concerns among residents during the COVID-19 pandemic were infecting their family (92.7%), disruption of social life (52.5%), interruption of residency training (49.7%), professional burnout (49.5%), transmitting COVID-19 to patients (42.4%), residency exams (42.4%), getting COVID-19 (41.3%), increased workload (40.7%), and uncertainty about the future (40.2%) (Table 3).

**Table 3:** COVID-19 characteristics of residents.

COVID-19 characteristics of residents		No.%
Does your job require you to treat patients diagnosed with COVID-19	Yes	434 (81.4)
Have you been isolated because of suspected SARS-CoV-2 infection	Yes	287 (53.8)
Have you been diagnosed with COVID-19	Yes	99 (18.6)
If yes, how many times have you been isolated		
One time		142 (49.5)
Two times		78 (27.2)
Three times		40 (13.9)
Four times		16 (5.6)
Five or more times		11 (3.8)
Which of the following is of most concern to you during the COVID-19 pandemic?		
Infecting your family		494 (92.7)
Disruption of social life		280 (52.5)
Interruption of residency training		265 (49.7)
Professional burnout		264 (49.5)
Transmitting COVID-19 to patients		226 (42.4)
Residency exams		226 (42.4)
Getting COVID-19		220 (41.3)
Increased workload		217 (40.7)
Future uncertainty		214 (40.2)
Inadequate protective equipment		161 (30.2)
Doing work that I usually would not do		145 (27.2)
Lack of knowledge		109 (20.5)
Financial problems		95 (17.8)
Inadequate training for infection control		94 (17.6)
Others: please specify		10 (1.9)

An association was observed between the residency level and the likelihood of isolation (P = 0.005). A post hoc analysis revealed a significant difference between R3 and R1 residents in terms of isolation (P = 0.005) (greater isolation in R3), and the difference was not significant between the other residency levels. Similarly, a significant association was found between residency level and the likelihood of being diagnosed with COVID-19 (P = 0.037). However, the difference among the different years of residency was significant only between R3 and R1 (P = 0.041) (greater isolation in R3). We found a significant association between COVID-19 diagnosis and male gender (P = 0.041). Additionally, a statistically significant association was found between resident specialties and the likelihood of being diagnosed with COVID-19 (P = 0.001). A post hoc analysis revealed a significant difference between internal medicine and family medicine (P = 0.030) (higher in internal medicine), internal medicine and other specialties (P = 0.046) (higher in internal medicine), emergency medicine and family medicine (P = 0.020) (higher in emergency medicine), and emergency medicine and other specialties (P = 0.025) (higher in emergency medicine).

A statistically significant association was present between resident specialties and treating patients diagnosed with COVID-19 (P < 0.001). However, the difference was significant between internal medicine and family medicine (P < 0.001) (higher in internal medicine), internal medicine and other specialties (P = 0.046) (higher in internal medicine), emergency medicine and family medicine (P = 0.020) (higher in emergency medicine), emergency medicine and other specialties (P < 0.001) (higher in emergency medicine), pediatric and family medicine (0.004) (higher in pediatric), pediatric and other specialties (P < 0.001) (higher in pediatric), family medicine and other specialties (0.007) (higher in family medicine), and surgery and other specialties (P < 0.001) (higher in surgery).

Residents who were on call were found to be significantly (P < 0.001) more exposed to patients with COVID-19. We found a significant association between COVID-19 diagnosis and smoking status, with a greater proportion of COVID-19 among nonsmokers than smokers (P = 0.044). Residents isolated because of suspected SARS-CoV-2 infection had a statistically significant association with anxiety symptoms (P = 0.004), as compared with non-isolated residents (Table 4).

**Table 4:** Relationships of sociodemographic characteristics and mental illness among residents with contact, isolation, and diagnosis of COVID-19.

Relationships of sociodemographic and mental illness among residents with contact, isolation, and diagnosis of COVID-19							
Category	N	Does the job require you to treat patients diagnosed with COVID-19		Have you been isolated because of suspected SARS-CoV-2 infection		Have you been diagnosed with COVID-19	
		Yes (%)	X^2^ value (P value)	Yes (%)	X^2^ value (P value)	Yes (%)	X^2^ value (P value)
**Residency Level**							
R1	114	89 (78.1%)	3.736 (0.443)	45 (39.5%)	14.65 (0.005)	14 (12.3%)	10.214 (0.037)
R2	131	105 (80.2%)		69 (52.7%)		23 (17.6%)	
R3	160	132 (82.5%)		97 (60.6%)		41 (25.6%)	
R4	99	81 (81.8%)		57 (57.6%)		14 (14.1%)	
R5	29	27 (93.1%)		19 (65.5%)		7 (24.1%)	
**Gender**							
Male	279	234 (83.9%)	2.314 (0.128)	147 (52.7%)	0.316 (0.574)	61 (21.9%)	4.189 (0.041)
Female	254	200 (78.7%)		140 (55.1%)		38 (15%)	
**Specialty**							
Int Med	71	69 (97.2%)	68.827 (0.000)	45 (63.4%)	7.730 (0.172)	21 (32.8%)	20.439 (0.001)
FM	142	105 (73.9%)		68 (47.9%)		18 (12.7%)	
EM	37	37 (100%)		23 (62.2%)		13 (35.1%)	
Surgical	141	121 (85.8%)		78 (55.3%)		30 (21.3%)	
Pedia	57	54 (94.7%)		33 (57.9%)		7 (12.3%)	
Others	85	48 (56.5%)		40 (47.1%)		10 (11.8%)	
**On call**							
Yes	379	324 (85.5%)	14.312 (0.000)	208 (54.9%)	0.566 (0.452)	71 (14.9%)	0.022 (0.882)
No	154	110 (71.4%)		79 (51.3%)		28 (14.9%)	
**Depression**							
Yes	303	251 (82.8%)	0.926 (0.336)	161 (53.1%)	0.143 (0.706)	57 (18.8%)	0.026 (0.871)
No	230	183 (79.6%)		126 (54.8%)		42 (18.3%)	
**Anxiety**							
Yes	311	245 (78.8%)	3.461 (0.063)	184 (59.2%)	8.497 (0.004)	56 (18%)	0.159 (0.690)
No	222	189 (85.1%)		103 (46.4%)		43 (19.4%)	
**Smoking**							
Yes	175	145 (82.9%)	0.353 (0.552)	94 (53.7%)	0.002 (0.966)	24 (13.7%)	4.069 (0.044)
No	358	289 (80.7%)		193 (53.9%)		75 (20.9%)	

Approximately two-thirds (66.9%) of the female residents and half (47.7%) of the male residents had depression symptoms. A statistically significant positive association was found between anxiety symptoms (P < 0.001) and depression symptoms (P < 0.001) in female residents compared with male residents. We additionally observed associations between residency level and depression symptoms (P < 0.001), and anxiety symptoms (P = 0.001). However, post hoc analyses indicated significant differences between R5 and other residency levels for both anxiety and depression symptoms (higher in R5). A statistically significant association was observed between resident specialties and depression symptoms (P < 0.001); the difference was significant between internal medicine and family medicine (P < 0.001) (higher in internal medicine), internal medicine and emergency medicine (P = 0.010) (higher in internal medicine), surgery and family medicine (P < 0.001) (higher in surgery), surgery and emergency medicine (P < 0.001) (higher in surgery), pediatric and family medicine (P = 0.002) (higher in pediatric), pediatric and emergency medicine (P = 0.035) (higher in pediatric), and other specialties and family medicine (P = 0.016) (higher in other specialties). Furthermore, we found statistically significant associations between resident specialties and anxiety symptoms (P < 0.001). The differences among specialties were significant between internal medicine and family medicine (P = 0.003) (higher in internal medicine), internal medicine and emergency medicine (P = 0.011) (higher in internal medicine), surgery and family medicine (P < 0.001) (higher in internal medicine), and surgery and emergency medicine (P = 0.003) (higher in internal medicine). An association was identified between monthly income and depression symptoms (P = 0.019). Post hoc analysis revealed a significant difference between residents whose monthly income was 15,000–20,000 SAR compared with >20,000 SAR (P = 0.017) (higher in 15,000–20,000). Statistically significant associations were found between anxiety symptoms (P < 0.001) or depression symptoms (P < 0.001) and on-call duty (Table 5).

**Table 5:** Prevalence of depression and anxiety, categorized by gender, residency level, specialty, income, and work duties.

Prevalence of depression and anxiety, categorized by gender, residency level, specialty, income, and work duties					
Item	N	Depression		Anxiety	
		Depression	X^2^ value (P value)	Anxiety	X^2^ value (P value)
**Gender**					
Male	279	133 (47.7%)	20.103 (0.000)	131 (47%)	31.283 (0.000)
Female	254	170 (66.9%)		180 (70.9%)	
**Residency level**					
R1	114	62 (54.4%)	24.405 (0.000)	69 (60.5%)	18.699 (0.001)
R2	131	71 (54.2%)		68 (51.9%)	
R3	160	83 (51.9%)		86 (53.8%)	
R4	99	58 (56.6%)		61 (61.6%)	
R5	29	29 (100%)		27 (93.1%)	
**Specialty**					
Int Med	71	48 (67.6%)	52.117 (0.000)	50 (70.4%)	33.508 (0.000)
FM	142	52 (36.6%)		63 (44.4%)	
EM	37	13 (35.1%)		14 (37.8%)	
Surgical specialties	141	104 (73.8%)		100 (70.9%)	
Pediatric	57	37 (64.9%)		38 (66.7%)	
Others	85	49 (57.6%)		46 (54.1%)	
**Monthly income in Saudi Riyals**					
<15,000	47	29 (61.7%)	7.963 (0.019)	30 (63.8%)	4.613 (0.100)
15,000–20,000	412	243 (59%)		246 (59.7%)	
>20,000	74	31 (41.9%)		35 (47.3%)	
**Living with family**					
Yes	399	222 (55.6%)	0.946 (0.331)	231 (57.9%)	0.135 (0.714)
No	134	81 (60.4%)		80 (59.7%)	
**On call as part of work**					
Yes	379	246 (64.9%)	34.734 (0.000)	247 (65.2%)	25.123 (0.000)
No	154	57 (37%)		64 (41.6%)	

Table 6 presents the independent correlates of depression symptoms by using univariate and multivariate analyses, and Table 7 shows similar results for anxiety. In a multivariate regression analysis, gender and residency level were independent correlates of anxiety symptoms. Female gender had an OR of 2.73 (95% CI 1.87–3.99, P = 0.001), and residency level (R5) had an OR of 5.48 (95% CI 1.19–25.16, P = 0.029). In contrast, the independent correlates of depression symptoms were gender (female) (OR: 2.25 [95% CI 1.54–3.29], P = 0.001) and specialty (family medicine) (OR: 0.42 [95% CI 0.20–0.88], P = 0.02) (Table 6).

**Table 6:** Prevalence of depression, categorized by gender, residency level, specialty, income, living with family and work duties, according to binary logistic regression analysis (univariate and multivariate analysis).

Item	Depression N (%)	Univariate			Multivariate		
		P Value	OR	[95% CI]	P Value	OR	[95% CI]
**Gender**							
Male	133 (47.7%)		ref				
Female	170 (66.9%)	0.000	2.02	1.56–3.16	0.000	2.25	1.54–3.29
**Residency level**							
R1	62 (54.4%)	0.893	ref				
R2	71 (54.2%)	0.98	0.99	0.42–1.17			
R3	83 (51.9%)	0.682	0.90	0.46–1.23			
R4	58 (56.6%)	0.54	1.19	0.60–1.82			
R5	29 (100%)	0.998	1354 …	–			
**Specialty**							
Int Med	48 (67.6%)	0.000	ref				
FM	52 (36.6%)	0.002	0.26	0.11–0.60	0.023	0.42	0.20–0.88
EM	13 (35.1%)	0.349	1.35	0.72–2.51			
Surgical	104 (73.8%)	0.749	0.89	0.42–1.85			
Pediatric	37 (64.9%)	0.203	0.65	0.34–1.3			
Others	49 (57.6%)	0.000	0.28	0.15–0.51			
**Monthly income in Saudi riyals**							
<15,000	29 (61.7%)	0.021	ref				
15,000–20,000	243 (59%)	0.719	0.89	0.48–1.7			
>20,000	31 (41.9%)	0.035	0.45	0.2–0.95			
**Living with family**							
No	81 (60.4%)	ref					
Yes	222 (55.6%)	0.331	1.22	0.82–1.8			
**On call as part of work**							
**No**	246 (64.9%)	ref					
**Yes**	57 (37%)	0.000	3.15	2.13–4.65			

∗Multicollinearity: no; overall accuracy: 61%; sensitivity: 61.1%; specificity: 60.9%; area under the curve (ROC): 72.0%; omnibus tests of model: p < 0.001; Hosmer–Lemeshow goodness of fit: P = 0.264; Nagelkerke R^2^: 22.0%.

**Table 7:** Prevalence of anxiety, categorized by gender, residency level, specialty, income, living with family and work duties, according to binary logistic regression analysis (univariate and multivariate analysis).

Item	Anxiety N (%)	Univariate			Multivariate		
		P Value	OR	[95% CI]	P Value	OR	[95% CI]
**Gender**							
Male	131 (46.9%)	ref					
Female	180 (70.9%)	0.000	2.75	1.92–3.93	0.000	2.72	1.87–3.95
**Residency level**							
R1	69 (60.5%)	0.009	ref				
R2	68 (51.9%)	0.176	0.70	0.42–1.17			
R3	86 (53.8%)	0.265	0.76	0.46–1.23			
R4	61 (61.6%)	0.871	1.05	0.60–1.82			
R5	27 (93.1%)	0.004	8.80	1.99–38.9	0.030	5.43	1.18–24.95
**Specialty**							
Int Med	50 (70.4%)	0.000	ref				
FM	63 (44.4%)	0.000	0.34	0.18–0.62			
EM	14 (37.8%)	0.001	0.26	0.11–0.59			
Surgical	100 (70.9%)	0.940	1.02	0.55–1.92			
Pediatric	38 (66.7%)	0.649	0.84	0.39–1.78			
Others	46 (54.1%)	0.04	0.49	0.26–0.96			
**Monthly income in Saudi Riyals**							
<15,000	30 (63.8%)	0.103	ref				
15,000–20,000	246 (59.7%)	0.585	0.84	0.45–1.57			
>20,000	35 (47.3%)	0.077	0.51	0.24–1.08			
**Living with family**							
No	80 (59.7%)	ref					
Yes	231 (57.9%)	0.714	0.93	0.62–1.38			
**On call as part of work**							
No	64 (41.6%)	ref					
Yes	247 (65.2%)	0.000	2.63	1.79–3.86			

**∗**Multicollinearity: no; overall accuracy: 61%; sensitivity: 61.1%; specificity: 60.9%; area under the curve (ROC): 72.0%; omnibus tests of model: p < 0.001; Hosmer–Lemeshow goodness of fit: P = 0.056; Nagelkerke R^2^: 18.3%.

## Discussion

The current study reports a high prevalence of depression symptoms (65.8%) and anxiety symptoms (58.3%) among medical residents in KSA, at levels higher than reported in several other countries.^16^ Female gender, residency level, and medical specialty were independent correlates of anxiety and depression symptoms.

In a study performed in France among residents and fellows of surgery during the COVID-19 pandemic, the prevalence of depression and anxiety symptoms was 40.8% and 35.9%, respectively.^17^ In another study among postgraduate trainees in Pakistan during the COVID-19 pandemic, the prevalence of depressive symptoms and generalized anxiety disorder was 26.4% and 22.6%, respectively.^18^ The overall prevalence of depression and anxiety symptoms was 25.3% and 13.7%, respectively, in a study performed among interns and residents across Panama during the COVID-19 pandemic.^16^ A previous similar study among residents in KSA by the SCFHS has shown comparable numbers, wherein more than half of the residents had depression and anxiety symptoms.^10^ However, the previous study did not assess the association between trainees’ medical specialties and the prevalence of depression and anxiety symptoms.

The prevalence of depression and anxiety symptoms among medical trainees was much higher than that in the general population (9.4% and 7.3%, respectively),^19^ and among university students (48.8% and 40.8%, respectively)^20^ and health care providers in KSA (55.2% and 51.4%, respectively) during the COVID-19 pandemic.^21^ The high workload of residents and the risk of infection from patients with COVID-19 may explain the high prevalence of both depression and anxiety symptoms.

The finding of an association between residents’ isolation due to COVID-19 and anxiety symptoms is in line with the results of a study among pediatric residents in the United States, which has indicated that social isolation negatively affects well-being.^22^ Isolation, in general, has a negative effect on well-being and includes social isolation from colleagues and an inability to engage in outdoor activities and social gatherings.^23^ In addition, social support is correlated with fewer mental health problems.^23^

The predisposition of female residents to depression and anxiety symptoms was demonstrated in the multivariate regression analysis and is consistent with findings from other studies. Depression and anxiety symptoms have been found to be significantly higher among women, younger respondents, and healthcare providers in a study performed among the general population in KSA during the COVID-19 pandemic.^24^ Anxiety symptoms have also been found to be significantly higher among female health care workers in KSA during the COVID-19 pandemic.^25^ Jordanian female health care professionals and university students have been found to be at higher risk of developing anxiety and depression symptoms.^26^ Similar findings have been observed among female postgraduate trainees in Pakistan.^18^ Our study revealed that the R5 residents had higher levels of anxiety and depression symptoms than other residents. This finding aligns with the results of other studies showing that senior trainees experience more anxiety and depression symptoms.^27^ The higher rate of anxiety symptoms among senior residents might be associated with a sense of greater responsibility and being concerned about their junior staff.

However, other studies have shown that junior residents have had more unsatisfactory outcomes, as well as high levels of depressive, anxiety, stress, and burnout symptoms during the COVID-19 pandemic.^28^^,^^29^ The difference in workload and the ability to handle stressors among junior residents may explain this finding. Senior residents usually have more responsibility and employment opportunities, whereas junior residents may have less experience and knowledge. In addition, residents who were on call as part of their work had more anxiety and depression symptoms.

The study showed that residents whose jobs included being on call were more exposed to patients with COVID-19. Being in contact with patients with COVID-19 probably predisposed the residents to anxiety and depression symptoms.^11^

The lower rate of SARS-CoV-2 infection among smokers than nonsmokers is consistent with results from other studies. However, behavioral factors or individual prior expectations and beliefs might have influenced the reporting. Thus, smokers might have felt that they were at higher risk of developing COVID-19 and consequently were more likely to report symptoms, thus exaggerating the apparent effects of smoking. Alternatively, nonsmokers, because they may be healthier in general, might be expected to be more sensitive to changes in their well-being and therefore more likely to report symptoms, thus underestimating the effects of smoking.^30^^,^^31^ However, we emphasize that other studies have found an association between smoking and a higher symptom burden in people testing positive, and a higher risk of hospitalization due to COVID-19 in smokers than nonsmokers.^31^

Finally, our study revealed that the most influential factor in being a resident diagnosed with COVID-19 was gender: male residents were diagnosed with COVID-19 at a higher rate than female residents, Moreover, being a nonsmoker rather than a nonsmoker was associated with a higher rate of COVID-19 diagnosis.

The current study provides new information on the effects of COVID-19 on mental health among medical trainees. It may enable new venues for the SCFHS and training programs to develop strategies to detect and treat these disorders in early stages and to implement a program for improving resident well-being, because it demonstrated a decrease in perceived stress and emotional exhaustion, and improvements in emotional intelligence and life satisfaction among residents.^32^^,^^33^ Anxiety and depression symptoms were highly prevalent among residents in KSA during the COVID-19 pandemic. Another strength of this study is that it covered several regions of KSA and used well-validated questionnaires to measure anxiety and depression symptoms.

Nevertheless, the observational cross-sectional nature of study precluded causal inferences and subjected the study to possible sampling bias. Additionally, this design makes investigating the temporal relationships between outcomes and risk factors difficult.

## Conclusion

The higher prevalence of anxiety and depression symptoms among residents in KSA is alarming. Future studies are needed to identify interventions to reduce the prevalence of anxiety and depression symptoms, and identify the causes of their high prevalence. In addition, an urgent need exists to establish a clinic in each health facility to address stress management and provide psychological support for health care workers.

## Source of funding

This research did not receive any specific grant from funding agencies in the public, commercial, or not for profit sector.

## Conflict of interest

The authors have no conflict of interest to declare.

## Ethical approval

The study was revised and approved by the institutional review board committee at King Saud University, College of Medicine, Riyadh, KSA, No. E−20-4755; Date: 16 December 2020.

## Authors contributions

HSA, HSM, and EAA performed literature review and discussion. AMA analyzed and interpreted data. FDA and ASB performed study review. All authors have critically reviewed and approved the final draft and are responsible for the content and similarity index of the manuscript.
