# Corrigendum to “Anxiety and depression symptoms among medical residents in KSA during the COVID-19 pandemic” [J Taibah Univ Med Sci 17(2) (2022) 192–202]

**DOI:** 10.1016/j.jtumed.2025.10.001

**Published:** 2025-10-24

**Authors:** Hossam S. Alawad, Hussein S. Amin, Eiad A. Alfaris, Abdullah M. Ahmed, Fahad D. Alosaimi, Ahmed S. BaHammam

**Affiliations:** a King Saud University Chair for Medical Education Research and Development, Department of Family and Community Medicine, College of Medicine, King Saud University, Riyadh, KSA; b Psychiatry Department, College of Medicine, King Saud University, Riyadh, KSA; c Department of Medicine, College of Medicine, University Sleep Disorders Centre, King Saud University, KSA; d The Strategic Technologies Program of the National Plan for Sciences and Technology and Innovation in the KSA, King Saud University, Riyadh, KSA

The authors regret:

In Table 2, the prevalence of depression is 56.8 %, which is the correct number. However, in the article, it is incorrectly stated as 65.8 %.

The correct sentences:

Abstract:

“The prevalence of depression and anxiety symptoms was 56.8 % and 58.3 %, respectively.”

"بلغت نسبة انتشار الاكتئاب 56.8 وأما القلق 58.3."

Results:

“The prevalence of depression and anxiety symptoms was 56.8 % and 58.3 %”

Discussion:

“The current study reports a high prevalence of depression symptoms (56.8 %) and anxiety symptoms (58.3 %) among medical residents in KSA”

The authors would like to apologise for any inconvenience caused.

